# Epidemiology of sclerotinia stem rot and efficacy of integrated control measures for soybean in Northeast China

**DOI:** 10.3389/fpls.2025.1679911

**Published:** 2025-12-01

**Authors:** Yichu Li, Jia Liu, Fengmei Shi, Ligong Ma, Yunhua Zhang, Liangbin Yu, Qinglin Meng

**Affiliations:** Institute of Plant Protection, Heilongjiang Academy of Agricultural Sciences, Harbin, Heilongjiang, China

**Keywords:** *Sclerotinia sclerotiorum*, Sclerotinia stem rot, white mold, soybean, disease severity index, fungicides

## Abstract

**Background/aims:**

Sclerotinia stem rot (SSR), caused by *Sclerotinia sclerotiorum*, is one of the most destructive fungal diseases affecting soybeans, and its effective management remains a challenge. This study aimed to investigate the epidemiology of SSR and to evaluate the efficacy of chemical fungicides, biocontrol agents (particularly low-risk, eco-friendly products), cultural practices, as well as to propose integrated strategies for SSR control in soybeans.

**Methods:**

Both small-scale and large-scale field trials were conducted in in Northeast China, the country’s largest soybean-producing region. The soybean varieties included Heinong 48, Kenfeng 16, Hefeng 50, Nongqingdou, Kendou 25, and Kendou 39.

**Results:**

The epidemiological study characterized the sclerotial germination dynamics and identified key factors influencing the disease severity index (DSI) and soybean yield. Assessment of low-risk, eco-friendly disease control products in small-scale field trials revealed that 6% oligosaccharins achieved the highest control efficacy of 70.0%. These findings informed the development of integrated control measures, which were then evaluated in scale-up field trials. Notably, these control measures significantly reduced disease incidence compared to control fields, demonstrating a disease control efficacy of 64.3–75.3%, alongside a yield increase of 5.7–14.7%. Subsequent implementation of the integrated measures achieved effective disease management, with a control efficacy of 56.41% and consistent yield improvements of 5.76–15.56%.

**Conclusions:**

Integrating disease-resistant variety selection, low-risk/eco-friendly chemical and biological agents, and cultural practices effectively manages SSR in soybean crops, significantly reducing DSI and increasing soybean yield in Northeast China. While these strategies may be applicable in other regions, optimal approaches may vary owing to regional differences and annual variations.

## Introduction

*Sclerotinia sclerotiorum* (Lib.) de Bary is a soilborne fungal pathogen that is responsible for the development of Sclerotinia stem rot (SSR), which is frequently referred to as white mold (Peltier et al., 2012; Hossain et al., 2023). *S. sclerotiorum* is challenging to manage for several reasons, such as its broad host range and its ability to produce long-lived sclerotia that can persist in soil for as long as five years, even under harsh environmental conditions (Hao et al., 2003; Duncan et al., 2006; Willbur et al., 2019). SSR is a potentially lethal disease characterized by white, cottony mycelial growth on stems, wilting, and the formation of black sclerotia (Jahan et al., 2022; Hossain et al., 2023). This disease affects a wide range of crops, including soybean—a globally vital source of protein and oil (Favaron et al., 2004; Li et al., 2024). As one of the most severe diseases of soybeans, SSR results in enormous yield and economic losses both in China and around the world (Yu et al., 2022). Over the past decade, there have been increasing reports of SSR severity in Northeast China, which primarily includes Heilongjiang, Jilin, and Liaoning Provinces, encompassing one of the three major “Black Soil” regions in the world (Liu et al., 2012). Northeast China serves as a major soybean-producing region, contributing to the nation’s largest soybean supply (Liu et al., 2021; Song et al., 2023; Zhao et al., 2023). In Northeast China, which accounts for over 40% of the nation’s soybean production, the crop plays a crucial role in ensuring food security and supporting the regional economy (Zhao et al., 2023). However, soybean production faces substantial challenges from fungal diseases, with SSR being one of the most destructive.

Currently, comprehensive epidemiological studies on SSR in Northeast China remain limited, which hinders the development of region-specific control strategies. The effective management of SSR relies on an integrated approach that combines cultural practices, chemical fungicides, and biocontrol agents (Peltier et al., 2012; Zeng et al., 2012; Smolińska and Kowalska, 2018; McCaghey et al., 2019; Webster et al., 2022, 2023). However, the efficacy of these measures varies due to environmental factors, pathogen adaptability, and farming practices. Although chemical control with fungicides can be effective, their application raises concerns about the development of resistance and environmental safety (Mueller et al., 2002; Wang et al., 2024). Given these challenges, a thorough understanding of SSR epidemiology—including spatiotemporal distribution, environmental drivers, and host–pathogen interactions—is essential for refining integrated disease management strategies. Previous studies in other countries or regions have demonstrated the influence of the soybean cultivar, soil moisture, planting density, and culture methods on SSR (Peltier et al., 2012; Smolińska and Kowalska, 2018; McCaghey et al., 2019; Wang et al., 2024; Debbink et al., 2025); however, regional validation is needed. Furthermore, the increasing adoption of conservation tillage and high-yielding soybean varieties in Northeast China may alter disease dynamics, necessitating updated control recommendations.

Meanwhile, biocontrol agents such as *Coniothyrium minitans* and *Trichoderma* spp, along with agronomic practices for managing SSR in soybean plants, have been extensively studied and show promise, but they require optimization for field-scale application. There is a research gap in combining this biofungicide with eco-friendly, biodegradable technology strategies, such as liquid mulch, which is composed of biodegradable materials that can minimize soil contamination and harm to non-target organisms (Vincent-Caboud et al., 2019; Menossi et al., 2021). These efforts are increasingly needed to address concerns about adverse environmental impacts and to meet the growing demand for organic soybean production (Vincent-Caboud et al., 2019).

Therefore, we conducted this study to investigate the epidemiology of SSR in Northeast China and to evaluate the effectiveness of chemical fungicides and biocontrol agents—especially low-risk and eco-friendly agents—and cultural methods. Based on the results, we proposed optimized management strategies tailored to regional conditions. The findings from this study may contribute to a broader understanding of *S. sclerotiorum* ecology and provide practical measures for disease management in one of China’s most important agricultural regions and beyond.

## Materials and methods

### Experimental field locations and soybean varieties

The trials were conducted in the experimental fields of the Bayi Agricultural University test base (Daqing, Heilongjiang, China) and at the Jiamusi Branch of the Heilongjiang Academy of Agricultural Sciences (Jiamusi, Heilongjiang, China) spanning the years of 2013 and 2014. The soil at the Daqing site was a loam-textured chernozem with a pH of 7.5. The soil at the Jiamusi site was a typical black soil (Mollisol), also with a loam texture, and had a pH ranging from 6.0 to 7.0. Supplemental sprinkler irrigation using groundwater was applied only during drought periods to prevent severe water stress.

Scale-up field trials were performed at the experimental farm of the Heilongjiang Academy of Agricultural Reclamation Sciences (Heilongjiang, China). The planting distances and their corresponding densities were as follows: 4 cm for 100,000 plants/ha, 3.3 cm for 300,000 plants/ha, and 2.9 cm for 350,000 plants/ha. The soybean varieties used in this study included Heinong 48, Kenfeng 16, Hefeng 50, Nongqingdou, Kendou 25, and Kendou 39.

### Disease incidence, disease severity index, and control efficacy

A large-area trial was conducted in 2013 with four large plots, each growing a different soybean variety and measuring 65 m². In 2014, each plot consisted of 24 rows with a row spacing (ridge width) of 65 cm and a row length of 7 m. Plots were arranged side-by-side and separated by six border rows on each side. Within each plot, disease assessments were conducted by randomly selecting three sampling sites. At each site, the experimental unit for data collection was a group of 100 consecutive plants within a single row. Disease severity was recorded for each plant based on the disease rating, using a scale of 0 to 9, as summarized in **Table 1**. This rating scale was adopted from previous studies (Boland and Hall, 1986; Song et al., 2009).

**Table 1:** Symptoms associated with different disease severity ratings using the scale adopted from Boland and Hall (1986) and Song et al. (2009).

Disease severity rating	Symptoms
0	None
1	Small disease spots on the stem; the plant exhibits normal growth.
3	Mild symptoms in the leaf axils and side branches during the early stage; lesions on the main stem are less than 3 cm in length, with a pod abortion rate less than 10%.
5	In the early stage, the main stem and side branches exhibit fungal hyphae and show water-soaked rot; in the later stage, lesions on the main stem ranging from 3 to 6 cm in length, appear pale at the site of the lesion, and have a pod abortion rate of 10–30%.
7	In the early stage, the main stem and side branches show extensive fungal growth, exhibiting severe water-soaked rot; in the later stage, lesions are pale, with dense sclerotia both inside and outside the main stem, measuring 6–15 cm in length, and a pod abortion rate of 30–50%.
9	Severe symptoms in the early stage, with the plant nearly dead; in the later stage, lesions on the main stem exceed 15 cm in length, with dense sclerotia both inside and outside the stem, and a pod abortion rate exceeding 70%.

Disease incidence was defined as the proportion of soybean plants infected by *S. sclerotiorum* within a surveyed population and was calculated using the following formula:


Disease incidence (%) = (Number of infected plants/Total plants surveyed) × 100.

Disease severity index (DSI) was a quantitative measure that assesses the overall severity of SSR within a surveyed population. The index was calculated using the following formula:


$$\begin{array}{c} \text{DSI}=[\sum (\text{Number of plants at each severity level}\\ \times \text{Corresponding level})/(\text{Total number of plants}\\ \times \text{Maximum severity level}]\times 100 \end{array}$$

The control efficacy for disease incidence was calculated as the percentage reduction in disease incidence compared to the untreated control, and the control efficacy for disease severity was calculated as the percentage reduction in DSI compared to the untreated control.

### Sclerotial germination of *S. sclerotiorum*

The sclerotial germination dynamics of *S. sclerotiorum* were monitored in two experimental fields: the Bayi Agricultural University test base in Daqing and the Jiamusi Branch of the Heilongjiang Academy of Agricultural Sciences in Jiamusi, Heilongjiang Province, China. Using a checkerboard sampling method, we established twelve 1-m² monitoring points across all infected plots in both locations. Germination assessments commenced at the early flowering stage of soybean and continued at 5-day intervals throughout the observation period.

### Biocontrol and chemical agents as well as other disease control products

This study evaluated a number of biocontrol and chemical agents as well as other disease control products for managing soybean SSR. The tested treatments were as follows: (1) The fungal biocontrol agent *C. minitans was* applied at three growth stages (sowing, seedling, and inter-tillage), with soil incorporation at rates of 562.5, 1125, and 2250 g/ha. The control efficacy of C. minitans was tested at three rates and three different application timings, resulting in a 3×3 factorial design; (2) The chemical control agents (40% dimethachlon WP and the plant activator Messenger) were administered via foliar spray. The initial application was conducted during the period from sclerotial germination through peak apothecium formation (late July to early August), followed by a second application one week later; (3) The low-risk/eco-friendly disease control agents **[**6% oligosaccharins aqueous solution (AS), *Bacillus subtilis* WP (1×10¹¹ CFU/g), and *Trichoderma* WP (2×10^9^ CFU/g)**]** were applied as a single treatment. The active ingredient of the 6% oligosaccharins AS is oligosaccharins, a plant resistance inducer that activates plant surface receptors and signal molecule transduction to enhance the disease tolerance of infected plants. All these agents were diluted with water according to their designated application rates and applied once during the flowering stage; and (4) The liquid mulch treatment assessed for physical barrier effects on the control of soybean SSR.

### Scale-up field experiments for efficacy of integrated control measures

Scale-up field trials were conducted at the experimental farm of the Heilongjiang Agricultural Reclamation Academy (Heilongjiang, China) to evaluate the efficacy of integrated control measures against soybean SSR, with sowing in May using the ridge triple-row cultivation technique at a density of 300,000 plants/ha and fertilization rates of 150 kg/ha diammonium phosphate, 30 kg/ha urea, and 25 kg/ha potassium sulfate. The following integrated control measures were tested in Treatment 1 (266.67 hectares (ha)): disease-resistant variety (Kendou 39), crop rotation (preferably with gramineous crops) or deep plowing (if the previous crop was legumes), reduced planting density, *C. minitans* biocontrol agent (1125 g/ha), liquid mulch (600× dilution, sprayed on ridges at the seedling stage), mechanical inter-row cultivation (pre-canopy closure to disrupt *S. sclerotiorum* apothecia), foliar spray of 6% oligosaccharins (600 g/ha in 600 kg water/ha), and dimethachlon (applied twice at 7-day intervals from disease onset). For comparison, Treatment 2 (26.67 ha) used conventional practices with Kendou 39 but no additional control measures. The control (26.67 ha) used the susceptible variety (Kendou 25) with no control measures. Disease incidence was assessed at five sampling points (20 m² each, serpentine pattern). The yield was determined at harvest by measuring the total weight of the harvested soybean from the scale-up plots and then converting it into kg/ha.

### Statistical analysis

Statistical analysis was conducted using Excel 2010, data processing system v7.05, and SPSS version 22. One-way analysis of variance (ANOVA) was performed to compare the DSI across three dependent assessments (I, II, III) among different treatment groups or conditions (various soybean varieties, planting densities, and fertilizer applications), followed by Duncan’s new multiple range test for *post-hoc* comparisons to determine statistical significance between treatments at the *P* < 0.05 and *P* < 0.01 levels. Multifactor analysis of variance (Multifactor ANOVA) was used to evaluate the effects of multiple factors, including different application times, application rates, and their interaction effects on Sclerotia germination, apothecia counts, and disease incidence.

## Results

### Factors influencing the incidence and severity of SSR, and yield of soybean

#### Soybean variety

The disease incidence and severity indices of SSR were evaluated in four soybean varieties, including Heinong 48, Kenfeng 16, Hefeng 50, and Nongqingdou, at three time points (**Table 2**). Heinong 48 exhibited resistance to SSR, with the lowest final disease incidence (11.34%) and severity index (8.07%), compared to the highly susceptible variety Kenfeng 16 (29.67% and 22.14%, respectively). We also evaluated the severity indices of SSR of Hefeng 50 and Heinong 48 in three independent assessments (**Table 3**). Heinong 48 displayed significantly lower disease indices in all three assessments (I, II, and III), with an average of 11.95% compared to 21.70% for Hefeng 50, indicating that Heinong 48 was less susceptible to SSR. Thus, the soybean variety had a significant impact on SSR, with disease onset occurring later in the resistant variety.

**Table 2:** Incidence and disease severity index of SSR in different soybean varieties.

Soybean variety	July 20		August 21		September 21	
	Incidence (%)	Disease severity index (%)	Incidence (%)	Disease severity index (%)	Incidence (%)	Disease severity index (%)
Heinong 48	0.00±0d	0.00±0d	8.36±0.04d	7.21±0.13d	11.34±0.11d	8.07±0.02d
Kenfeng 16	7.33±0.35a	2.89±0.10a	23.33±0.07a	11.82±0.05a	29.67±0.12a	22.14±0.09a
Hefeng 50	3.56±0.37c	0.98±0.05c	10.67±0.06c	9.77±0.06b	14.72±0.10c	10.72±0.07c
Nongqingdou	6.27±0.29b	1.42±0.01b	11.96±0.08b	8.05±0.34c	17.33±0.09b	11.17±0.03b

The trial was conducted in the experimental fields of the Bayi Agricultural University test base (Daqing, Heilongjiang, China), with soybean as the previous crop. Random sampling was conducted at three growth stages. For each plot, three sampling points were selected, with 100 plants evaluated per point. SSR, Sclerotinia stem rot.

**Table 3:** Severity indices of SSR in two soybean varieties.

Soybean variety	Disease severity index				Statistical significance	
	I	II	III	Mean ± SE	5% level	1% level
Hefeng 50	20.15	25.28	19.67	21.7±1.80	a	A
Heinong 48	14.04	10.24	11.57	11.95±1.11	b	B

The trial was conducted in the experimental fields of the Bayi Agricultural University test base (Daqing, Heilongjiang, China). Different letters (a, A, b, B) indicate different levels of statistical significance. Hefeng 50 and Heinong 48 are two predominant soybean varieties cultivated in China’s major soybean-producing area (Heilongjiang, China). The average disease severity of SSR was calculated from three independent assessments (I, II, and III). Statistical significance was determined at the 5% and 1% probability levels. SSR, Sclerotinia stem rot.

#### Planting density

Three planting density levels, including low (250,000 plants/ha), medium (300,000 plants/ha), and high (350,000 plants/ha), were evaluated for their effect on the severity of SSR in two soybean trial fields in Daqing and Jiamusi (soybean varieties Hefeng 50 and Hefeng 55, respectively). As shown in **Table 4**, a higher planting density was associated with an increased disease severity at both trial locations. In addition, significant differences were observed among all three density levels (250,000, 300,000, and 350,000 plants/ha) in both trial locations. These results indicate that SSR may worsen with a higher planting density, suggesting that, in high-risk regions, reducing the planting density may help mitigate disease incidence and minimize yield loss.

**Table 4:** Effect of the planting density on the disease severity index of SSR at two soybean trial field locations (Daqing and Jiamusi).

Planting density	Disease severity index				Statistical significance	
	I	II	III	Mean ± SE	5% level	1% level
Daqing 250,000/ha	11.38	8.92	10.56	10.29±0.72	c	B
300,000/ha	14.24	20.44	15.43	16.70±1.90	b	AB
350,000/ha	19.51	21.96	23.86	21.78±1.26	a	A
Jiamusi 250,000/ha	25.78	27.11	26.67	26.52±0.39	c	B
300,000/ha	32.22	32.44	28.67	31.11±1.22	b	AB
350,000/ha	38.00	35.33	38.67	37.33±1.02	a	A

Soybean varieties: Hefeng 50 (Daqing) and Hefeng 55 (Jiamusi). The trials were conducted in the experimental fields of the Bayi Agricultural University test base (Daqing, Heilongjiang, China) and the Jiamusi Branch of Heilongjiang Academy of Agricultural Sciences (Jiamusi, Heilongjiang, China). Planting density is defined as the number of soybean plants cultivated per hectare. The average disease severity of SSR was calculated from three independent assessments (I, II, and III). Statistical significance was determined at the 5% and 1% probability levels. The soil at the Daqing site was a loam-textured chernozem with a pH of 7.5. The soil at the Jiamusi site was a typical black soil (Mollisol), also with a loam texture, and had a pH ranging from 6.0 to 7.0. Supplemental sprinkler irrigation using groundwater was applied only during drought periods to prevent severe water stress. SSR, Sclerotinia stem rot; ha, hectare.

#### Fertilizer application

The effects of three fertilizer types (N, P, and K) and their application rates on the severity of SSR were evaluated. As summarized in **Table 5**, there were significant differences in the DSI of SSR among soybean variety Hefeng 45 treated with different fertilizer types. The DSI significantly increased with higher nitrogen (N) and phosphorus (P) application rates, respectively, whereas the potassium (K) application rates had no significant effect on the DSI.

**Table 5:** Effects of different fertilizers and their application rates on the disease severity index of SSR.

Fertilizer application (kg/ha)	Disease severity index				Statistical significance	
	I	II	III	Mean ± SE	5%	1%
N20+P150+K50	18.00	20.89	19.78	19.56±0.84	g	E
N40+P150+K50	21.11	20.00	21.78	20.96±0.52	fg	DE
N60+P150+K50	22.22	20.89	22.22	21.78±0.44	efg	CDE
N80+P150+K50	23.11	25.78	27.78	25.56±1.35	bcd	BCD
P130+N80+K50	22.22	25.11	23.56	23.63±0.84	def	CDE
P150+N80+K50	26.22	29.56	29.56	28.45±1.11	bc	B
P170+N80+K50	28.22	26.44	32.22	28.96±1.71	b	B
P190+N80+K50	34.44	34.67	35.56	34.89±0.34	a	A
K30+N80+P150	22.22	27.33	24.00	24.52±1.50	de	BCD
K50+N80+P150	24.22	27.56	24.89	24.56±1.02	bcd	BCD
K70+N80+P150	27.56	25.78	24.44	25.93±0.91	bcd	BC
K90+N80+P150	25.78	24.00	25.78	25.19±0.59	cde	BCD
0 + 0+0	26.44	21.11	26.00	24.52±1.71	de	BCD

Soybean variety: Hefeng 45. Fertilizers applied: urea (N), diammonium phosphate (P), and potassium sulfate (K). The trial was conducted in the experimental fields of the Jiamusi Branch of Heilongjiang Academy of Agricultural Sciences (Jiamusi, Heilongjiang, China). The average disease severity of SSR was calculated from three independent assessments (I, II, and III). Statistical significance was determined at the 5% and 1% probability levels. SSR, Sclerotinia stem rot.

#### Soil moisture

**Figure 1:**
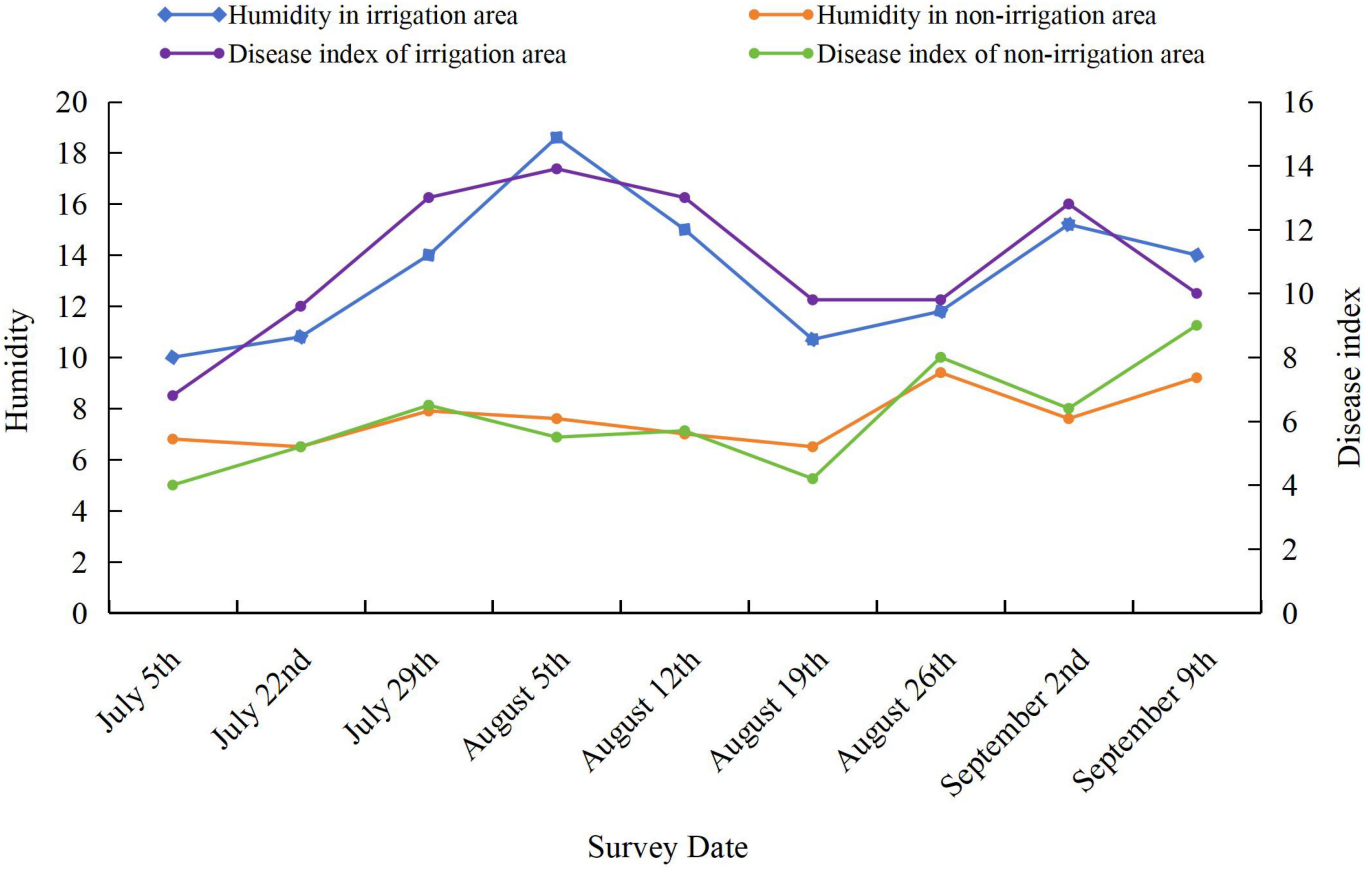
Effect of soil moisture on the disease severity index of Sclerotinia stem rot (SSR). Soybean variety: Nongqing. The experimental field was divided into two areas: a nonirrigated area (natural rainfall) as a control and an irrigated area (watered every 7 days). The planting density was maintained at 350,000 plants/ha. The soil moisture was measured during each assessment, and the unit was percentage (%), defined as the amount of water in the soil relative to the weight of the dry soil. The disease severity index was calculated based on three independent assessments.

The effect of soil moisture on the severity of SSR was evaluated by growing soybean plants in either an irrigated area (watered every 7 days) or a nonirrigated area (natural rainfall only). As shown in **Figure 1**, the severity of SSR differed significantly between the irrigated and nonirrigated areas. A higher soil moisture content in the irrigated area led to more severe SSR.

#### Continuous cropping, crop rotation, and cultivation practices

As shown in **Table 6**, continuous soybean cropping significantly increased the incidence of SSR, while rotating soybean crops with corn crops reduced its incidence. Furthermore, the two cultivation methods (double-row ridge versus triple-row ridge) exhibited no significant differences in either the incidence or DSI of SSR.

**Table 6:** Effect of continuous cropping, crop rotation, and cultivation practices on the incidence and disease severity index of SSR.

Preceding crop and cultivation method	Disease incidence (%)	Disease severity index	Statistical significance
Preceding crop Soybean	40.2	N/A	N/A
Corn	19.4	N/A	N/A
Cultivation method Double-row ridge	31.4	11.22	aA
Triple-row ridge	32.2	13.84	aA

Trials were conducted at Keshan Farm (Heilongjiang, China) using the soybean cultivar Beidou 14. The preceding crop refers to the crop grown in the same field immediately prior to soybean cultivation. The disease incidence was calculated as the percentage of soybean plants infected by *S. sclerotiorum*. Double-row ridge: two parallel rows of soybeans planted on each ridge; Triple-row ridge: three parallel rows of soybeans per ridge; SSR, Sclerotinia stem rot.

### Sclerotial germination of *S. sclerotiorum* at different field locations

**Figure 2:**
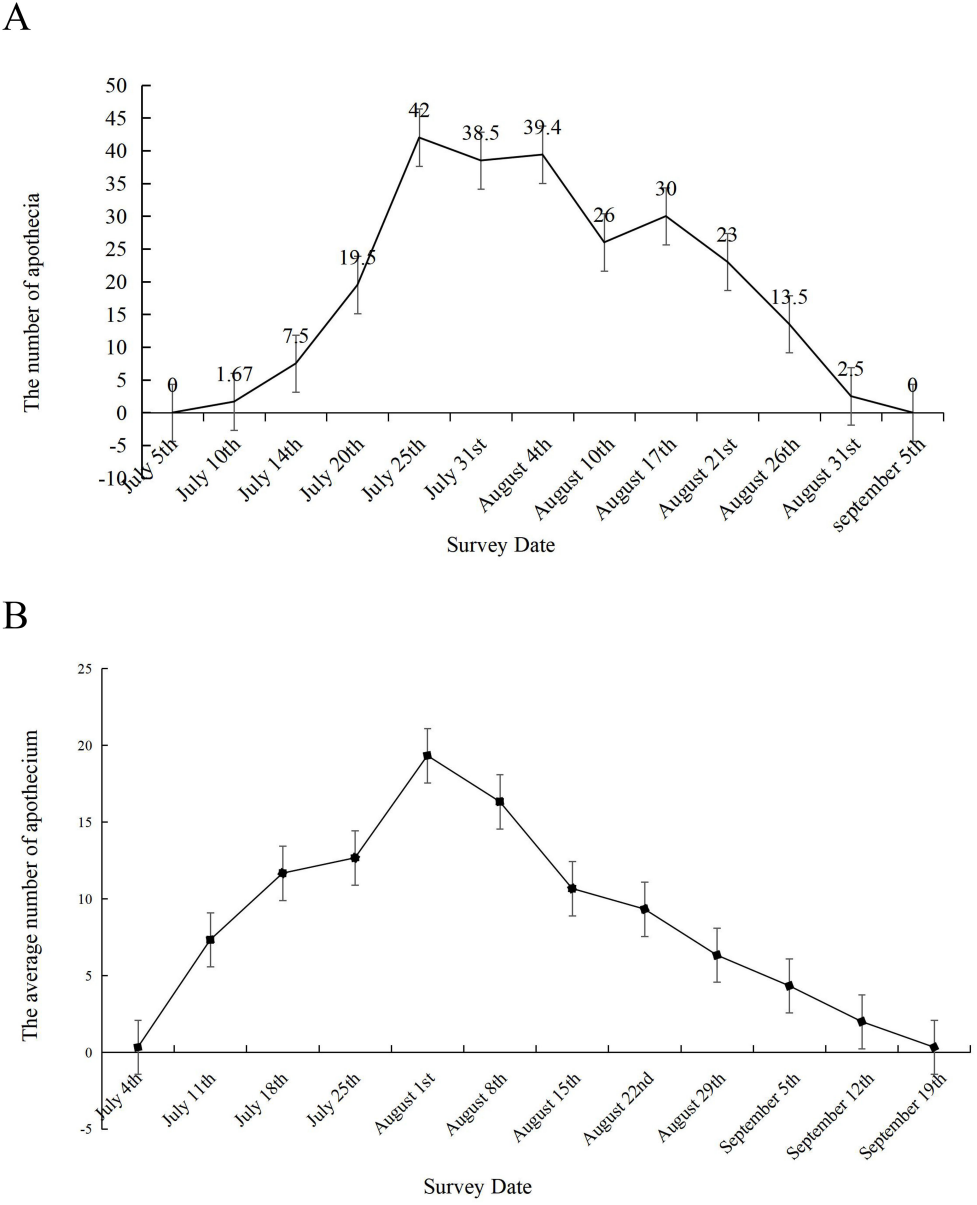
Sclerotial germination dynamics of *S. sclerotiorum* in two soybean-producing regions. The sclerotial germination dynamics in **(A)** Daqing and **(B)** Jiamusi, two soybean-growing regions of Heilongjiang Province, China. For each location, a grid sampling method (1-m² quadrats each) was employed to select 12 sampling areas. Germination monitoring began at the initial flowering stage of soybean and continued at 5-day intervals. Both locations displayed similar patterns of sclerotial germination, including germination initiation, peak germination period, and entire duration.

The sclerotial germination dynamics of *S. sclerotiorum* were monitored in two soybean growing regions of Heilongjiang Province, China (Daqing and Jiamusi). In Daqing (**Figure 2A**), germination initiation occurred on July 10, with only 20 apothecia detected across 12 sampling areas. The peak germination period spanned from July 20 to August 21, with the maximum number of apothecia observed on July 25. The germination rates remained consistent until August 4, followed by a progressive decline. By August 31, only sparse apothecia were detectable. The entire germination period lasted approximately 52 days. Similar patterns of sclerotial germination, including germination initiation, peak germination period, and total duration, were observed in Jiamusi (**Figure 2B**).

### Assessment of biological agents, chemical agents, and other products in the control of SSR

#### Biocontrol agent *C. minitans*

Trials were conducted to evaluate the application rate and timing (applied at the sowing, seedling, and cultivation stages) of *C. minitans* on SSR control (**Table 7**). Application rates of *C. minitans* at 1125 g/ha and 2250 g/ha during the sowing and seedling stages resulted in a significant decrease in both apothecia and sclerotia germination, along with an increase in control efficacy. Specifically, the effectiveness in reducing disease incidence reached 59.3% for the 1125 g/ha application rate and 60.2% for the 2250 g/ha application rate when applied at the sowing stage. Both application rates exhibited a nearly equivalent control efficacy, with the higher rate of 2250 g/ha not yielding any further improvement in control efficacy. From an economic perspective, the application of 1125 g/ha at the sowing stage may provide the best cost–benefit ratio. In contrast, when applied after the seedling stage and in the inter-tillage stage, no clear trend was observed in sclerotia germination or apothecia formation across the three application rates. Overall, the sowing-stage application demonstrated the highest control efficacy, followed by the seedling-stage application, while the inter-tillage application was the least effective.

**Table 7:** Effects of the *C. minitans* application rate and timing on the control of soybean SSR.

Application timing	Application rate (kg/ha)	Apothecia count	Control efficacy (%)	Sclerotia germination	Control efficacy (%)	Disease incidence	Control efficacy (%)
Sowing stage	0.56	790.5±4.92	37.0	2359.5±196.46	37.8	6.4	45.8
	1.13	420±1.73	66.6	1135.5±136.17	70.0	4.8	59.3
	2.25	499.5±1.80	60.2	1314±225.06	65.3	4.7	60.2
	**Average**		**54.6**		**57.7**		**55.1**
Seedling stage	0.56	970.5±0.87	22.7	2950.5±210.39	22.2	7.8	33.9
	1.13	844.5±6.07	32.7	2590.5±379.39	31.7	7.4	37.3
	2.25	655.5±1.80	47.8	2550±97.00	32.7	6.1	48.3
	**Average**		**34.4**		**28.9**		**39.8**
Inter-tillage	0.56	589.5±1.80	53.1	2340±254.86	38.3	9.2	22.0
	1.13	775.5±12.61	38.2	2770.5±103.36	26.9	9.4	20.3
	2.25	940.5±15.77	25.1	3105±275.23	18.1	8.2	30.5
	**Average**		**38.8**		**27.8**		**24.3**
Blank control	Control (untreated)	1255.5		3790.5		11.8	-

*C. minitans* is a fungal biocontrol agent against *S. sclerotiorum*. *C. minitans* was applied as the soil treatment after mixing with soil. Trials were conducted in Heilongjiang, China, using the soybean variety Nongqing No. 1. The disease incidence was calculated as the percentage of soybean plants infected by *S. sclerotiorum*. The control efficacy was determined as the percentage reduction compared to the untreated control. SSR, Sclerotinia stem rot; ha, hectare. The bold values represent the averages of three control efficacy percentages (%).

Key findings from the multifactor ANOVA are summarized in **Table 8**. The application of *C. minitans* at the inter-tillage stage with a rate of 2.25 kg/ha resulted in the highest number of Sclerotia germinations. In contrast, application of *C. minitans* at the sowing stage led to significantly fewer sclerotial germinations. Both application rate and timing, as well as their interaction, had a statistically significant effect on apothecia counts. Additionally, the highest effect on disease incidence was observed when *C. minitans* was applied at the sowing stage. Conversely, application during the inter-tillage stage had the least effect on disease incidence.

**Table 8:** Multifactor ANOVA of the effects of *C. minitans* application timing and rate on sclerotial germination, apothecia count, and disease incidence.

Dependent variable	Source of variation	df	Mean square	*F*-value	*P*-value
Sclerotia Germination	Application Time	2	160,001.52	2986.09	< 0.001
	Application Rate	2	27,433.33	511.99	< 0.001
	Time × Rate	4	127,227.67	2374.44	< 0.001
Apothecia Counts	Application Time	2	3,731,877.75	73.53	< 0.001
	Application Rate	2	336,263.25	6.63	0.007
	Time × Rate	4	781,167.00	15.39	< 0.001
Disease Incidence	Application Time	2	29.70	192.32	< 0.001
	Application Rate	2	4.89	31.68	< 0.001
	Time × Rate	4	0.72	4.68	0.009

#### Low-risk and eco-friendly disease control products

A number of low-risk chemical agents and disease control products were evaluated for their effects on controlling SSR, and the resulting data are summarized in **Table 9**. The trial results showed that, under a disease incidence of 36.9% in the untreated control group, treatment with 6% oligosaccharins achieved a control efficacy of 70.0%, followed by *B. subtilis* with an efficacy of 49.6%. ANOVA indicated that the disease incidence with 6% oligosaccharins treatment was significantly less than those of the other disease control products. However, no significant differences were observed among the other treatments. Moreover, all treatment groups exhibited a significantly reduced disease incidence compared to the untreated control group.

**Table 9:** Efficacy of low-risk/eco-friendly disease control products against SSR.

Product	Disease incidence (%)				Control Efficacy (%)	Yield (kg/ha)	Yield increase rate (%)
	I	II	III	Mean ± SE			
6% Oligosaccharins AS	10.8	11.8	9.8	10.8±0.58c	70.0	2001	7.9
*B. subtilis* WP	21.4	18	15.1	18.17±1.82b	49.6	1972	6.4
*Trichoderma* WP	19.9	26.4	20.2	22.17±2.12b	38.4	1968	6.1
40% Dimethachlon WP	20.9	28.3	20.2	23.13±2.59b	35.8	1903	2.6
Messenger	18	21.4	19.2	19.53±1.0b	45.8	2106	13.6
Liquid mulch	19.7	24.7	16.3	20.23±2.44b	43.8	1708	-7.9
Control (untreated)	36.9	35.5	35.5	35.97±0.47a	–	1854	–

Small-scale field trials were conducted in Heilongjiang, China, using the soybean variety Nongqing No. 1. The disease incidence was defined as the percentage of soybean plants infected by *S. sclerotiorum*. The control efficacy was calculated as the percentage reduction in disease incidence compared to the untreated control. The yield increase rate was determined as the percentage gain in yield compared to the untreated control. SSR, Sclerotinia stem rot; ha, hectare.

### Efficacy of scale-up field trials in the control of SSR

A scale-up field trial was conducted to evaluate the efficacy of *C. minitans* and oligosaccharins for the control of SSR in soybeans. As presented in **Table 10**, under conditions where the incidence of soybean SSR in the control area was 15.2%, the control efficacy was 74.2% for the biocontrol agent *C. minitans*, 75.3% for the low-risk chemical agent 6% oligosaccharins, and 64.3% for the combination of 6% oligosaccharins + *C. minitans*. All three treatments significantly reduced the disease incidence compared to the untreated control; however, there was no significant difference among the three treatments.

**Table 10:** Efficacy of *C. minitans* and oligosaccharins alone or in combination for the control of soybean SSR in a scale-up field trial.

Agent	Disease incidence (%)				Control efficacy (%)	Yield (kg/ha)	Yield increase rate (%)
	I	II	III	Mean ± SE			
*C. minitans*	3.1	5.6	3.1	3.93 ± 0.83b	74.2	1861.6	5.7
6% Oligosaccharins	6.3	2.5	2.5	3.77 ± 1.27b	75.3	2001.1	13.6
6% Oligosaccharins + *C. minitans*	5.0	3.8	7.5	5.43 ± 1.09b	64.3	2021.4	14.7
Control (untreated)	23.8	11.3	10.6	15.23 ± 4.29a	–	1761.8	–

The scale-up field trial was carried out in Heilongjiang, China, using the soybean variety Heinong 51. The treatments were as follows: *C. minitans—*application rate, 1125 g/ha; application method, soil incorporation and surface application; field area, 1.33 ha; oligosaccharins*—*application rate, 600 g/ha; application method, foliar spray at the flowering stage; field area, 13.33 ha; *C. minitans* and oligosaccharin*—*the combined application methods were the same as the individual treatments above; field area, 1.33 ha; Untreated control*—*field area, 2.67 ha. The disease incidence was defined as the percentage of soybean plants infected by *S. sclerotiorum*. The control efficacy was calculated as the percentage reduction in disease incidence compared to the untreated control. The yield increase rate was determined as the percentage gain in yield compared to the untreated control. SSR, Sclerotinia stem rot; ha, hectare.

Based on the small-scale field trial results, various practical measures were recommended for the control of soybean SSR according to the cultivation stage (**Table 10**).

Scale-up field trials of integrated practical measures in the control of SSR showed that Treatment 1 (integrated control measures + disease-resistant soybean variety) significantly reduced the disease incidence to 13.62%, showing a highly significant difference compared to the untreated control, with a control efficacy of 56.41%. In contrast, Treatment 2 (disease-resistant soybean variety) achieved a lower control efficacy of 16.70%, though it still exhibited a highly significant difference in disease incidence compared to the untreated control field (**Table 11**). Notably, Treatment 1 increased the average yield by 15.56% compared to the untreated control field. These results demonstrate that the integrated management strategy effectively controlled SSR while substantially enhancing soybean productivity (**Tables 11**, **12**).

**Table 11:** Efficacy of integrated practical measures for soybean SSR control in a scale-up field trial.

Treatment	Disease incidence (%)						Control efficacy (%)	Statistical significance	
	1	2	3	4	5	Mean ± SE		5%	1%
1	11.21	13.69	16.27	14.80	12.11	13.62±0.91a	56.41	a	A
2	20.21	27.42	28.54	27.44	26.52	26.03±1.49b	16.70	b	B
Control (untreated)	25.38	35.24	36.27	30.24	29.14	31.25±2.01c	—–	c	C

Large-scale field trials were conducted at the experimental farm of the Heilongjiang Academy of Agricultural Reclamation Sciences (Heilongjiang, China). The treatments were as follows: Treatment 1, integrated control measures + disease-resistant soybean variety *Kendou 39*; Treatment 2, disease-resistant soybean variety *Kendou 39 (no additional control measures)*; and Control, untreated, disease-susceptible soybean variety *Kendou 25*. Statistical significance was determined at the 5% and 1% probability levels. SSR, Sclerotinia stem rot.

**Table 12:** Effects of integrated practical measures on soybean production in a scale-up field trial.

Treatment	Yield (kg)						Yield increase rate (%)
	1	2	3	4	5	Mean ± SE	
1	3023.25	3231.36	3319.77	3338.20	2965.47	3175.61±76.71a	15.56
2	2756.32	2864.18	2978.31	2634.25	2946.17	2835.85±63.34b	5.76
Control (untreated)	2660.37	2672.51	2653.17	2663.26	2757.26	2681.31±19.24b	—–

Large-scale field trials were conducted at the experimental farm of the Heilongjiang Academy of Agricultural Reclamation Sciences (Heilongjiang, China). The treatments were as follows: Treatment 1, integrated control measures + disease-resistant soybean variety *Kendou 39*; Treatment 2, disease-resistant soybean variety *Kendou 39* (no additional control measures); and Control, untreated, disease-susceptible soybean variety *Kendou 25*.

Collectively, large-scale field trials were conducted across approximately 18.7 hectares, demonstrating a disease control efficacy of 64.3–75.3% with a corresponding yield increase of 5.7–14.7% (**Table 10**). Subsequent implementation of the integrated control measures across nearly 120 hectares maintained effective disease management, with a disease control efficacy of 56.41%, while achieving consistent yield improvements of 5.76–15.56% (**Tables 11**, **13**).

**Table 13:** Recommended integrated practical measures for controlling soybean SSR.

Stage	Recommended practical measures
Pre-sowing: Field selection	Practice crop rotation; select fields previously planted with cereal crops
Pre-sowing: Field preparation	Carry out deep plowing (especially in fields previously planted with legumes or sunflowers) to reduce pathogen viability
Pre-sowing: Variety selection	Choose the following resistant soybean varieties: Hefeng 55, Heinong 48, Heinong 44, Heinong 60, or Suinong 10
Fertilization	Reduce nitrogen fertilizer and increase organic fertilizer in heavily infested fields
Sowing	Reduce the planting density appropriately (use the lower limit of the recommended density for the corresponding cultivar
Sowing	Apply the biocontrol agent *C. minitans* (750–1,125 g/ha) mixed with sieved fine soil, evenly incorporated into seed furrows
Flowering	Use a foliar spray of 6% oligosaccharins solution, with an application rate of 600 g/ha
Inter-tillage	Perform mechanical cultivation before canopy closure to disrupt *S. sclerotiorum* apothecia formation
Post-inter-tillage	Apply liquid mulch (600× dilution) mechanically on ridges
Post-canopy closure	At disease onset (late July), apply 40% dimethachlon WP (1,050 g/ha, 600 L/ha) twice at 7-day intervals [FRAC Group Code: 2 (Class: Dicarboximide); medium-high risk in the FRAC Dicarboximide Guidelines for Resistance Management].

SSR, Sclerotinia stem rot; ha, hectare.

## Discussion

This study investigated the epidemiology of SSR in Northeast China, the nation’s largest soybean-producing region, and evaluated the efficacy of chemical fungicides, biocontrol agents, and cultural practices in managing this devastating and hard-to-control disease affecting soybeans. Through small- and large-scale field trials, the key findings were as follows: (1) the epidemiological study characterized the sclerotial germination dynamics of *S. sclerotiorum* and identified critical factors influencing SSR severity and yield loss; (2) small-scale field trials evaluated the effects of low-risk, eco-friendly disease control products for managing SSR in soybean, suggesting that 6% oligosaccharins achieved the highest control efficacy of 70.0% among all tested control products; (3) practical integrated control measures were developed based on the small-scale field trials of epidemiological studies and efficacy measurement of control products; and (4) implementation of integrated control measures in scaled-up field trials achieved a disease control efficacy of 56.41% with yield increases of 5.76–15.56%. Collectively, these results demonstrate that integrating chemical, biological, and cultural control measures effectively reduced the SSR severity while enhancing the yield, providing a practical management strategy to mitigate the impact of SSR on soybean production.

The epidemiological study characterized the sclerotial germination patterns, including germination initiation, peak germination period, and total duration. We uniformly placed laboratory-cultivated sclerotia to infect the experimental fields, with consistent sources and quantities of sclerotia placed. Therefore, the differences in germination numbers among locations may be attributed to varying soil moisture and climatic conditions. We are inclined to believe that soil moisture and climatic conditions were the primary factors influencing the variation in the number of sclerotia germinating. Our results have important implications for disease management; for example, specifically knowing when germination initiates and peaks may help optimize the timing of fungicide applications and cultural control measures (e.g., tillage, irrigation adjustments). The early detection of germination enables preemptive actions to reduce widespread infection.

Several key factors influencing SSR severity were identified, including the soybean variety, planting density, soil moisture, fertilizer application, and cultivation practices. The results demonstrate that SSR incidence and severity increased with a higher planting density, greater nitrogen (N) and phosphorus (P) application rates, and elevated soil moisture levels. Additionally, the cultivation methods (double-row versus triple-row ridge systems) showed no significant differences in disease incidence or severity. The soybean variety significantly affected the disease incidence and severity, with resistant varieties exhibiting delayed disease onset. Our observations align with previous studies showing that the soybean cultivar plays a critical role and that high levels of moisture favor disease development (Grau and Radke, 1984; Workneh and Yang, 2000; Kurle et al., 2001; Debbink et al., 2025). The DSI varied significantly across soybean cultivars, suggesting that genetic resistance exerts a role in SSR management. However, complete resistance is rare in commercial soybean varieties, necessitating supplementary control measures. Our results, together with those from others (Grau and Radke, 1984; Workneh and Yang, 2000; Kurle et al., 2001), suggest that in high-risk regions, implementing control measures such as reducing the planting density and selecting disease-resistant varieties may help mitigate SSR and minimize yield loss.

It is worth noting that we evaluated the effects of low-risk, eco-friendly disease control products for managing SSR in soybeans. The small-scale field trial results demonstrate that 6% oligosaccharins achieved the highest control efficacy of 70.0% among all tested control products. This finding highlights oligosaccharins as a promising, sustainable tool for SSR management in soybeans, aligning with global trends toward green agriculture and the growing demand for organic soybean production. In addition, the application of eco-friendly control products, such as liquid mulch, may address the concerns about adverse environmental impacts (Vincent-Caboud et al., 2019). Liquid mulch, typically composed of biodegradable materials, can minimize soil contamination and harm to nontarget organisms (Vincent-Caboud et al., 2019; Menossi et al., 2021). Nevertheless, future research is needed to optimize application methods and to expand trials.

Based on the results of the small-scale trials, we proposed integrated practical measures for controlling soybean SSR, and their efficacy and beneficial impact on soybean production were assessed in large-scale field trials. Two experimental treatments and one control were tested: Treatment 1 consisted of integrated control measures in combination with the disease-resistant soybean variety *Kendou 39*; Treatment 2 included the disease-resistant soybean variety *Kendou 39* without any additional control measures; and the Control group consisted of the untreated, disease-susceptible soybean variety *Kendou 25*. It was noteworthy that the combined approach in Treatment 1—utilizing a disease-resistant soybean variety, timely applications of control products, and optimal cultural methods—significantly reduced the disease incidence to 13.62%, with a significant difference compared to the untreated control, with a control efficacy of 56.41%. In contrast, Treatment 2, which used only the disease-resistant soybean variety, achieved a lower control efficacy of 16.70%, though it still displayed a highly significant difference in disease incidence compared to the untreated control. Interestingly, this translated into a yield increase of 5.7–15.56% compared to the untreated control field. These results demonstrate that the integrated management strategy effectively controlled SSR while substantially enhancing soybean productivity. Furthermore, the findings support the notion that integrated disease management not only controls diseases but also enhances the overall productivity. This finding also suggests that disease-resistant varieties can provide some level of control and that there is a need to develop varieties with greater disease resistance through genetic modification (El-Baky and Amara, 2021; Zhao et al., 2022). However, relying solely on resistant varieties may not suffice to effectively manage complex diseases, like SSR. Their effectiveness could be greatly enhanced when combined with additional control measures, as demonstrated by our results (control efficacy of 56.41% versus 16.70% for Treatment 1, which utilized a disease-resistant soybean variety, timely applications of control products, and optimal cultural methods, compared to Treatment 2, which used only the disease-resistant soybean variety).

It must be mentioned that our study may have some limitations. For instance, although the integrated control strategy proposed in this study proved effective in controlling SSR, its broader application may face challenges. The variability of environmental conditions across regions (e.g., soil moisture, temperature, humidity) could affect control consistency and limit generalizability to other agroecological regions (Matheron and Porchas, 2005). In the scale-up experiments, the unbalanced design and confounding factors could have introduced management bias. To achieve better and more effective SSR management, future research efforts should focus on developing soybean varieties with enhanced SSR resistance through host resistance breeding and genetic modification. Additionally, adopting precision agriculture tools, such as weather-based models to predict sclerotial germination (Willbur et al., 2018) and to optimize fungicide timing, could be beneficial for managing SSR.

In conclusion, this study demonstrates that a combination of disease-resistant variety selection, low-risk/eco-friendly chemical and biological agents, and cultural practices effectively manages SSR in soybean crops, leading to a reduced disease severity and an increased yield in Northeast China. These measures have potential applicability in other areas of Northeast China. It is important to note that, due to regional specificity and annual variation, the optimal measures may differ across regions and environmental conditions.

## Data Availability

The raw data supporting the conclusions of this article will be made available by the authors, without undue reservation.
